# Track and trace: how soil labelling techniques have revealed the secrets of resource transport in the arbuscular mycorrhizal symbiosis

**DOI:** 10.1007/s00572-022-01080-7

**Published:** 2022-05-21

**Authors:** Stephanie J. Watts-Williams

**Affiliations:** The Waite Research Institute and School of Agriculture, Food and Wine, The University of Adelaide, PMB 1, Glen Osmond, South Australia, 5064 Australia

**Keywords:** Arbuscular mycorrhizal fungi, Hyphal compartment, Mycorrhizal uptake pathways(s), Plant nutrition, Quantum dot tracking, Radioisotope labelling

## Abstract

Arbuscular mycorrhizal (AM) fungi colonise plant roots, and by doing so forge the ‘mycorrhizal uptake pathway(s)’ (MUP) that provide passageways for the trade of resources across a specialised membrane at the plant–fungus interface. The transport of nutrients such as phosphorus (P), nitrogen and zinc from the fungus, and carbon from the plant, via the MUP have mostly been quantified using stable or radioactive isotope labelling of soil in a specialised hyphae-only compartment. Recent advances in the study of AM fungi have used tracing studies to better understand how the AM association will function in a changing climate, the extent to which the MUP can contribute to P uptake by important crops, and how AM fungi trade resources in interaction with plants, other AM fungi, and friend and foe in the soil microbiome. The existing work together with well-designed future experiments will provide a valuable assessment of the potential for AM fungi to play a role in the sustainability of managed and natural systems in a changing climate.

## Introduction

Arbuscular mycorrhizal (AM) fungi associate with the roots of most terrestrial plants, including many important agricultural crops. The AM association is based on a trade of resources between plant and fungus–photosynthate C from the host plant, and nutrients acquired in the soil such as phosphorus (P), nitrogen (N) and zinc (Zn) from the fungus.

**Fig. 1:**
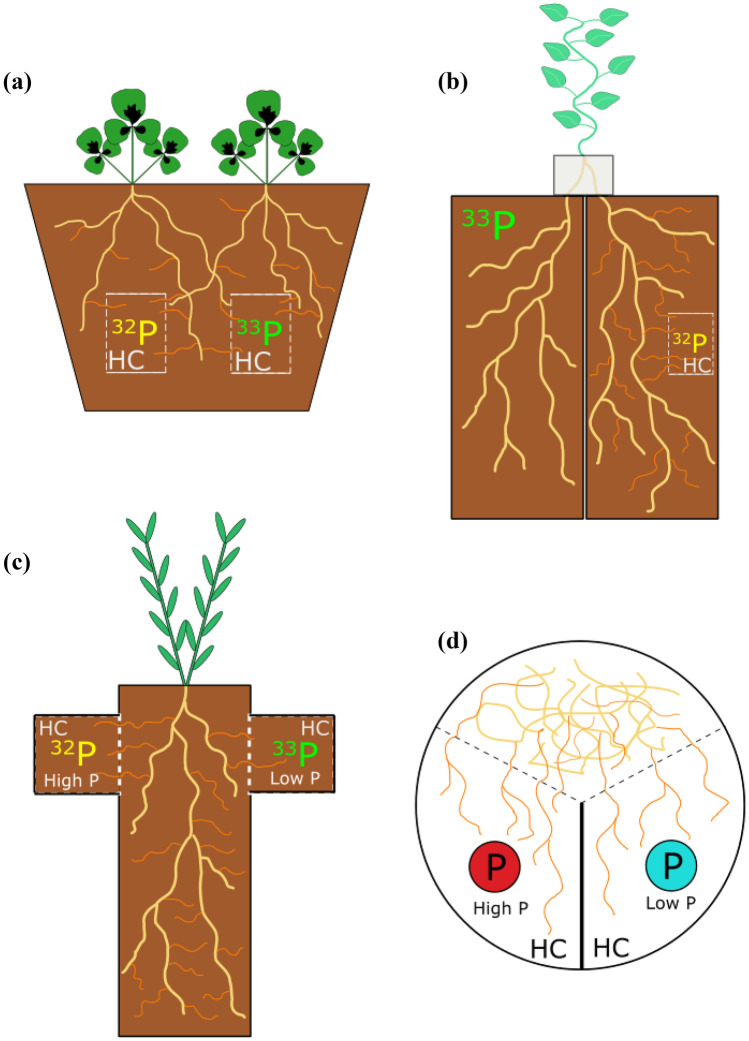
Schematic diagrams of four experimental designs to quantify the activity of the arbuscular mycorrhizal (AM) pathway(s) of uptake: a regular pot containing a specialised hyphal compartment (HC) with labelled soil, e.g. Svenningsen et al. (2018) (**a**), a split-pot which can be used with or without a HC if one side contains AM fungal inoculum and isotope and the other does not, e.g. Grønlund et al. (2013) (**b**), a cross-pot with hyphal compartment side arm(s) containing isotope and differential P supply, e.g. Cavagnaro et al. (2005) (**c**), and a split-plate that allows for detailed and precise tracing experiments including with quantum dots, e.g. Whiteside et al. (2019) (**d**). Each of these examples uses dual labelling of phosphorus but also could be used with a single isotope/quantum dot or hyphal compartment; the design is dependent on the research question

For almost as long as there has been research on AM fungal associations, there have been questions about what, how and where resource exchange occurs between the plant and colonising fungus. Isotope labelling techniques were employed to demonstrate the proof-of-concept for bidirectional resource transport (Ho and Trappe 1973; Cooper and Tinker 1978). This technique has two important components: a ‘hyphal compartment’ filled with soil that is guarded by mesh that allows penetration by fungal hyphae, but not roots; and labelling of the compartment’s soil with a stable or radioactive isotope that later can be detected and quantified in plant tissues (Fig. 1) (Schweiger and Jakobsen 2000).

Once the capacity for AM fungi to transport resources via the mycorrhizal uptake pathway(s) (MUP) was established, it was followed by an abundance of contextual information on plant and fungus functional diversity, and soil and environmental factors, and included the use of radioisotopes in the field (Chiariello et al. 1982; Jakobsen et al. 1992; Schweiger and Jakobsen 1999). The vast amount of groundwork that was laid paved the way for isotope labelling to answer additional intriguing questions, and made the link with advances in molecular biology, particularly the expression of AM-specific *phosphate transporter* (*PT*) genes.

As scientists grapple with modern-day challenges, including the increasing pressure to attain food and water security in a changing climate, and to find alternative sources of plant nutrients for agriculture as limited, non-renewable sources (especially of P) become increasingly scarce, there is a growing role for AM fungi in building resilience (Thirkell et al. 2017; Field et al. 2021). The recent published (last two decades) experimental work covered in this review has made important contributions to the above challenges, and others. There is great potential for future isotope labelling studies to target solutions to these global challenges in both managed and natural ecosystems (see Future perspectives), although there are limitations to the technique in both controlled-environment and field experiments that need to be considered.

## The role of AM fungi in a changing climate

The way that plants and fungi grow, develop, acquire and allocate resources will change substantially in the future, and studies that incorporate predicted future temperature, rainfall and atmospheric carbon dioxide (CO_2_) concentrations into their designs are invaluable. Resource trade is crucial to AM symbiosis, so it is necessary to consider how this trade will function given some resources are predicted to increase (photosynthates through elevated CO_2_ concentration) while others may decrease (plant-available P and N), or be dependent on geographic location (water), in the future. The studies described in this section give us a valuable glimpse into how activity of the MUP and resource trade will function amid elevated global temperatures and CO_2_ concentrations, and variable water availability.

### Will elevated CO_2_ stimulate AM fungi and the MUP?

The predicted increase in atmospheric CO_2_ concentrations may confer a ‘fertilisation effect’ on many species of plants, including some important crop species (Müller et al. 2014). There are concerns, however, about a counter-productive ‘dilution effect’ of nutrients in plant tissues because of increased plant biomass without a proportional increase in nutrient uptake, and whether that could be mitigated by AM fungal contributions to nutrient acquisition (He and Nara 2007; Thirkell et al. 2022). It has been hypothesised that AM fungi will drive the nature of the fertilisation effect due to interactions with N availability (Terrer et al. 2016) and P availability (Treseder 2004).

Gavito et al. (2002) presented the first work tracing the MUP under elevated CO_2_ conditions (697 ppm). In pea (*Pisum sativum*; C_3_) plants, P uptake via the MUP was reduced at elevated CO_2_, despite the shoots generally being larger and having greater total P content than at ambient CO_2_. This was followed by a study on the interactive effects of elevated CO_2_ (688 ppm) and soil temperature (10 and 15 °C) on pea plants colonised by *Funneliformis caledonium* or an AM fungal population from field soil (Gavito et al. 2003). Extraradical hyphae only developed in the hyphal compartment containing ^33^P at 15 °C, so MUP activity could not be compared between the temperature treatments. However, at 15 °C, there were no effects of atmospheric CO_2_ concentration on MUP activity, which led the authors to conclude that future elevated soil temperature, rather than CO_2_, will affect AM function and P transport. Jakobsen et al. (2016) also reported no effect of elevated CO_2_ on MUP activity in both legume (*Medicago truncatula*; C_3_) and grass (*Brachypodium distachyon*; C_3_) species. Rather, the MUP activity, which contributed up to 80% of shoot P in *M. truncatula* and 70% in *B. distachyon*, was highly dependent on soil P availability and plant species.

Recent experiments in wheat (*Triticum aestivum* L.; C_3_) and barley (*Hordeum vulgare* L.; C_3_) extended the investigation into plant–fungal resource trade under elevated CO_2_ (800 ppm) with stable (^15^N, ^14^C) alongside radioisotope (^33^P) labelling (Thirkell et al. 2020, 2021). The authors reported that P transport via the MUP was highly driven by cultivar identity but not CO_2_ in wheat; in one barley cultivar (Moonshine), there was significantly greater contribution to shoot P via the MUP under elevated compared to ambient CO_2_. N transport via the MUP was unaffected by CO_2_ concentration in all the barley and wheat cultivars studied. Likewise, the amount of C transported to the colonising AM fungus (*Rhizophagus irregularis*) was not influenced by atmospheric CO_2_ concentration. However, more N was delivered via the MUP in the Moonshine than in the Riviera barley cultivar. Given the contrasting results for the N and P MUPs in these experiments, it would be interesting for future studies using isotopes of N and P simultaneously to test hypotheses regarding how the ratio of available N to P in soil influences the activities of the respective MUPs.

A summary of the limited number of available labelling studies is that AM fungi will continue to effectively colonise plants under elevated CO_2_ and temperature conditions, and will contribute to plant P and N uptake. However, the activity of the MUP may not be affected by CO_2_ and there was little evidence of AM fungi being ‘fed’ by the extra assimilated C from these labelling studies. However, a meta-analysis of non-labelling studies reported that mycorrhizal abundance (arbuscular and ectomycorrhizal) increased by 47% under elevated CO_2_ (Treseder 2004), although this was highly modulated by nutrient (N and P) availability. Under future CO_2_ concentrations, the host plant may be able to acquire additional nutrients via root growth when ‘fertilised’ by CO_2_, but the activity of the MUP may not make an extra contribution to total shoot P or N, except in certain cultivars. Further studies that identify genotypes of crops that can enhance MUP activity under elevated CO_2_ conditions (e.g. Moonshine barley) will be important.

### New evidence of a role for AM fungi in drought stress resilience

Drought (water) stress resilience is considered one of several non-nutritional benefits conferred to plants by AM fungi. Many experiments have demonstrated that AM plants accumulate more biomass and nutrients, and transpire more under water-limited conditions than their non-AM controls (Augé 2001), and have the ability to regulate specific aquaporin genes (Krajinski et al. 2000; Uehlein et al. 2007). This has led to an understanding that in general, AM fungi confer water use efficiency in their host plant and will help plants tolerate lower water availability in the future.

Symanczik et al. (2018) used labelled N to show that more N was transported via the MUP when sorghum (*Sorghum bicolor*) plants were colonised by the endemic *Rhizophagus arabicus* than by *Rhizophagus irregularis*. The shoot ^15^N concentration (%) was greater in well-watered than drought-stressed condition with *R. arabicus*, and not different between watering treatments with *R. irregularis*. Similarly, Püschel et al. (2021) grew *M. truncatula* with *R. irregularis* along a continuum of water availability and traced the P transported via the mycorrhizal and direct pathways of uptake. Contribution to shoot P from the immediate (root-adjacent) source of P was greater in the mycorrhizal plants at medium and low moisture regimes. However, the distant source of P, contained within a root-exclusion compartment accessible to AM hyphae, appeared in shoots only with high water availability treatment.

The first direct evidence for AM fungal transport of water from extraradical hyphae to the host plant was reported from isotope-labelled water (^18^O) tracing in a pre-print article (Kakouridis et al. 2020). Wild oat (*Avena barbata*) plants were colonised by *Rhizophagus intraradices*, hyphae of which crossed a 3.2 mm air gap into a hyphal compartment to access the isotope-labelled water. The water transpired by the AM-colonised plants contained significantly more ^18^O than the non-AM control plants, lending evidence that the direct effect(s) of AM fungi on plant water relations are potentially substantial and may contribute to plant drought resilience.

## The mycorrhizal pathway of P uptake for efficient agricultural systems

AM fungal propagules are commonplace in agricultural soil systems, except in cases where antagonistic management practices may have affected their abundance, diversity and/or functionality (Zhang et al. 2019). AM fungi also associate with the vast majority of broadacre and horticultural crop species, including cereals, pulses and many vegetables—canola (*Brassica napus*) and sugar beet (*Beta vulgaris*) being the notable exceptions. Yet, there is ongoing debate in the AM research community as to whether there is a meaningful role for AM fungi in increasing nutrient uptake, yields, and thus food security in agriculture (Ryan and Graham 2018; Rillig et al. 2019). Furthermore, the staggering number of commercial AM fungal products now available on the market, coupled with their unregulated quality controls, and thus inconsistent or negative results, has led to distrust and hesitancy to incorporate AM fungi as a legitimate factor in agronomic decision-making (Hart et al. 2018; Thomsen et al. 2021; Salomon et al. 2022). The more information we can generate on how different crops, soils, management practices and commercial products affect the activity of the MUP, the closer we are to being able to provide realistic and reliable advice on how to manage the plant–fungal association agronomically.

### The what, where and who of P transport via the MUP

Historically, P uptake via the MUP has generated the most interest and studies using isotope labelling, despite there being no available stable isotopes of P for this research—only radioactive P. Perhaps the most important work from recent years is that by Smith et al. (2003), which overturned the idea that growth responses (difference in biomass between +AM plant and -AM controls) could be used as a proxy for activity of the mycorrhizal pathway of P uptake. Previously, a plant that was non-responsive in biomass (e.g. tomato; *Solanum lycopersicum*) or was not highly colonised by AM fungi was presumed to have received little P via the MUP. To the contrary, Smith et al. (2003) calculated that the MUP could contribute a substantial proportion of shoot P in the three plant species grown—tomato, flax (*Linum usitatissimum*), and *M. truncatula*. Following this work, it was discovered that the MUP could contribute up to 70% of P in rice (*Oryza sativa*) (Yang et al. 2012), and up to 60% in maize (*Zea mays*), although contribution by the MUP varied with maize genotype (Sawers et al. 2017; Chu et al. 2020). Recently, Zhang et al. (2021) investigated the potential trade-off between P uptake via the direct (plant only) versus the mycorrhizal pathway in three maize varieties using ^32^P labelling. They reported that plant preference for the mycorrhizal pathway prevailed when soil P availability was suboptimal (Olsen P of 8 mg P kg^−1^), but not when soil P was deemed low (4.5 mg P kg^−1^) or high (50 mg P kg^−1^) for the plant.

Another important point elucidated by isotope labelling is that expression of mycorrhiza-specific *phosphate transporter* (*PT*) genes (e.g. *MtPT4*, *OsPT11*) and direct pathway *PT* genes do not generally serve as a proxy for activity of the mycorrhizal and direct pathways of uptake, respectively. Generally, the activity of the MUP and expression of mycorrhiza-specific *PT* genes are repressed as soil P availability increases (e.g. in tomato; Nagy et al. (2009)), but Grønlund et al. (2013) showed in a split-pot, dual-isotope experiment that the activity of the MUP can repress direct pathway *PT* gene expression throughout the root system, but suppress direct pathway P transport only locally. Using a similar setup, Watts-Williams et al. (2015a, b) reported that there was limited systemic influence of a patch of AM-colonised roots on direct pathway *PT* expression in *M. truncatula*, but a repressive effect on the activity of the direct pathway in distant non-colonised roots. External P availability exerts strong influence over the two P uptake pathways and *PT* expression, but the interplay between the two pathways across a root system, as represented by isotope uptake or *PT* expression, is dynamic and complex.

Along with host plant functional diversity, the MUP is highly influenced by AM fungal diversity. Ravnskov and Jakobsen (1995) first showed that MUP transport of P was high with one species of AM fungus and not with another, which was then also confirmed with different communities of AM fungi from field soils (Jakobsen et al. 2001). Munkvold et al. (2004) grew cucumber plants with 24 isolates from the phylum Glomeromycota and found massive variation in the amount of P transported (from 0 to 75 kBq plant^−1^), and found MUP activity could be correlated with extraradical hyphal length density. Cavagnaro et al. (2005) reported similar AM fungal functional diversity, with *Gigaspora rosea*, *Funneliformis mosseae* and *R. irregularis* taking up 0.06, 0.2, or 0.3 µg P m^−1^ hyphae, respectively.

That the identity of the host plant and colonising AM fungus are substantial drivers of MUP activity has important consequences for implementation of commercial AM fungal products into agriculture. Results of a given product are likely to be dependent on many factors, such as crop, species of AM fungi in the product and already in the soil, and soil P concentration, among others. For example, three wheat cultivars were grown in intact soil cores from the field that were supplemented with a *R. irregularis* inoculum product, and the potential of the product was tested by tracing P and N uptake via the MUP (Elliott et al. 2021). There was no effect of the AM fungal product on wheat aboveground biomass, nor MUP activity in two of the wheat varieties, but in the third variety, MUP activity decreased. Similar studies with other products, soils and crops will be important for determining the future of AM fungal inocula in agriculture.

### Other belowground abiotic interactions—organic sources, fungicides and heavy metals

To realise sustainable agriculture goals, we will need to leverage the potential of alternative sources of P fertiliser including from organic waste products that are not readily plant-available. It is imperative that we understand how this will affect AM associations with plants, and the capacity for the MUP to function. We also need information on how AM fungi are affected by fungicides, pesticides and micronutrients, as reliance on these increases in agricultural management.

Early studies using radioisotopes of P demonstrated that AM fungi can, in the presence of other microbes, use organic, plant-derived sources of P (Joner and Jakobsen 1994, 1995). Proof-of-concept that AM fungi can mineralise organic sources of P and transport them to the host plant in the absence of other soil microbes was demonstrated subsequently using ^32^P (Joner et al. 2000). There has been increased interest in how AM fungi perform when supplied with organic sources of P (Ngo et al. 2021), with the idea that substitution of typical rock phosphate with organic waste sources may provide options for sustainable fertiliser use. Mackay et al. (2017) demonstrated with ^33^P labelling that P uptake via the MUP was active when fertilised with dried sewage sludge, but not with incinerated sewage sludge.

The effect of agricultural fungicide applications on the function of AM fungi has long been a topic of interest; the widely-used benzimidazole antifungal carbenzadim has a pronounced suppressive effect on MUP activity at high rates (Kling and Jakobsen 1997; Schweiger and Jakobsen 1998), but a stimulatory effect on the MUP at low rates (Schweiger et al. 2001). Use of municipal wastewater in agricultural practice is a proposed mechanism to conserve freshwater reserves in the future, but wastewater can contain numerous contaminants, including various azole antifungals from medical sources. Environmentally relevant contamination levels of three commonly prescribed azole antifungals reduced the transport of P via the MUP to wheat plants (Sallach et al. 2021); such contaminants could hamper our ability to exploit municipal wastewater, and AM fungi for P uptake, in the future.

Zinc is an essential plant micronutrient and important for human nutrition; it has been demonstrated previously with ^65^Zn that the MUP of Zn is active (Bürkert and Robson 1994; Mehravaran et al. 2000; Jansa et al. 2003). The contribution of Zn via the mycorrhizal pathway relative to the direct pathway was first quantified in tomato shoots by Watts-Williams et al. (2015a, b) and found to contribute up to 24% of the Zn to shoots when grown in Zn-deficient soil. Subsequently, the MUP was demonstrated to contribute 28 and 12% of Zn to the grain portion of wheat and barley (Coccina et al. 2019), which demonstrated that AM fungi can potentially contribute to the accumulation of Zn for nutritious cereal products. Given the relatively low (but critical) plant requirement for micronutrients such as Zn compared to N and P, these are substantial proportions of plant Zn arriving via the MUP.

Other trace elements briefly have been studied through radioisotope labelling, but none were given the same attention as Zn. A multitracer experiment (i.e. multiple radioisotopes applied to the same hyphal compartment) investigated whether 14 plant trace elements could be transported to marigold (*Tagetes patula*) plants by the AM fungus *Claroideoglomus etunicatum* (Suzuki et al. 2001); while the radioisotopes of Zn, sodium, selenium, rubidium, strontium and ytrium were transported in significantly greater amounts to the mycorrhizal than the non-mycorrhizal marigold plants, the other elements were either not detected in plant tissues, or found in similar quantities in both treatments. Neither the iron nor the manganese radioisotope was detected in any plants, which points to an issue with the experimental design (e.g. quantity of isotope added, uptake, or detection in tissues) rather than a true result, as we would expect both elements to reach detectable levels in plants. Further studies in other important crop plants would be useful to form a complete picture of AM fungal contribution to Zn, and other micronutrient, uptake.

## Interactions with friend and foe in the mycorrhizosphere

Underground biotic interactions involving AM fungi are notoriously difficult to capture and quantify. For that reason, studies that have used isotope labelling in combination with donor-receiver plant set-ups or next generation sequencing of the soil microbiome have delivered invaluable insights into the ‘hidden half’ and its effects—promotive or suppressive—on the activity of the MUP.

### The terms of plant–fungus resource trading between partners and across networks

The association between AM fungus and plant hinges on the trade of resources. As in society, the trade of resources sometimes involves a power struggle, or individuals that cheat the system for their own gain. A series of experiments have successfully uncovered this ‘underground stock market’ between plants and AM fungi, in partnership and in networks with other plants, using isotopes and novel tracing techniques.

By using split plates with hyphal compartments, Kiers et al. (2011) demonstrated with isotopes that host plants can discriminate between less- and more- ‘cooperative’ AM fungal partners, and can even differentially provide increased C to the ‘best’ fungus. However, the fungus can also preferentially supply P to roots that provide the most C. By similar methods, Fellbaum et al. (2012) found that plant supply of C to the fungus is necessary to trigger fungal N transport in return. In a split-root microcosm system, when two different AM fungal species were in competition for host plant C, the fungus that provided more P to the host plant, and in turn received more C from the host plant than its competitor fungus (Argüello et al. 2016), highlighting the potential importance of diversity in AM fungal communities.

An exciting advance in MUP tracing is the advent of quantum dots, which allow tracking of fluorescent nanoparticles attached to a resource such as organic nitrogen (N) (Whiteside et al. 2009) or rock phosphate (Whiteside et al. 2019). Using quantum dots of different colours to differentiate between P that originated from a ‘rich’ or a ‘poor’ patch, Whiteside et al. (2019) showed that *R. irregularis* has the capacity to respond to resource variation by changing how it distributes, stores and shifts resources within its network. van't Padje et al. (2021) went further to show using quantum dots that *R. irregularis* can successfully manipulate the value of P and its allocation to the host plant depending on fungal access to P sources (i.e. a simulated P ‘boom’ or ‘crash’). However, the P demands of young roots can influence how *R. irregularis* distributes P to host roots and how P is stored in fungal structures (van't Padje et al. 2021). It is important to note that the diameter of the quantum dot nanoparticles attached to nutrient sources (e.g. tagged rock phosphate) is substantially large compared to that of an untagged orthophosphate anion. This likely has implications for how the quantum dot-tagged P source moves in vivo compared to an untagged P source. The effects of the physical attributes of quantum dot-tagged nutrient sources on their transport and movement in plant and fungal systems are discussed in Whiteside et al. (2019), but characterisation of the specific uptake pathways of quantum dot-tagged apatite by AM fungi, and subsequent transport to and movement in plants, requires further fundamental characterisation.

Common mycorrhizal networks (CMNs) are belowground connections between individual plants, even of different species, facilitated by the mycelia of their colonising AM fungi (see Bonneau et al. 2019 for a recent book chapter on the subject). Experimental work on CMNs using isotope labelling has demonstrated that P and N transfer between plants connected by a CMN can be substantial, for example between subterranean clover plants colonised by *F. mosseae* (Mikkelsen et al. 2008), and between flax and sorghum plants colonised by *R. irregularis* or *F. mosseae*—to the benefit of flax more than sorghum (Walder et al. 2012). Merrild et al. (2013) showed that competition between plants can be amplified by CMNs, with intact hyphal networks favouring an established plant over a new seedling, unless the supply of C from the established plant ceased (e.g. by grazing or senescence). Lekberg et al. (2010) proposed a mycocentric view of CMNs after discovering that donor plants sent C (as lipids) to a C-limited receiver plant, but the C remained in the fungal tissue. Nevertheless, roots of non-C-limited plants had greater arbuscule densities and received 10 times more isotope-labelled P than those of C-limited plants. Taken together, the ‘terms of trade’ for C, P and N between neighbouring plants joined by a CMN is dependent on the species of plants involved, the proximity and establishment status of the plants, and the supply of C to the colonising fungus.

### Other biotic interactions in the soil—hostiles and helpers

The soil microbiome plays host to a consortium of organisms that interact with AM fungi, plants, or both. Isotope labelling has allowed researchers to quantify whether interactions with an individual species or microbial community have positive or negative effects on AM function based on the effect on MUP activity.

Using dual-P isotope labelling, Svenningsen et al. (2018) determined that the MUP in *M. truncatula* was suppressed when the external mycelia sourced P from a patch of non-sterilised soil, compared to a patch of sterilised soil. Further experiments demonstrated that suppression of AM fungal function exists along a continuum in cultivated soils, with more suppressive soils having high abundances of acidobacteria. When they followed this finding in a set of non-cultivated soils, Cruz-Paredes et al. (2019) found the biotic suppressive effect was similarly present. Taken together, the works conclude that the P-transport function of AM fungi in both cultivated and natural soils is strongly modulated by the presence and abundance of specific soil microbes, most likely bacterial.

In contrast, other soil microbes have the ability to promote the activity of the MUP; evidence for this comes from an isolated strain of plant growth-promoting bacterium (*Streptomyces* sp. W77) tested by Battini et al. (2017), and two rhizosphere yeasts (*Cryptococcus flavus* and *Candida railenesis*) tested by Sarabia et al. (2018). Other bacterial strains tested by Battini et al. (2017) promoted the growth of extraradical mycelium or overall P uptake by an AM plant, but not activity of the MUP per se. De Jaeger et al. (2011) reported increased uptake of ^33^P by extraradical mycelia, but disruption of transport to the host plant, in the presence of the saprotrophic fungus *Trichoderma harzianum*, a biocontrol agent used against plant–fungal pathogens.

Recently, recycling and uptake of organic sources of N also have been associated with AM fungi (Bukovská et al. 2021), which was attributed to cooperation with microbial helpers (Rozmoš et al. 2021). The AM fungus *R. irregularis* was able to obtain significantly more ^15^N from an organic source (chitin) when both the protist *Polysphondylium pallidum* and bacteria *Paenibacillus* sp. were present in the hyphosphere. Microbial interactions likely influence AM fungal uptake of other sources of N (and P), and are deserving of further investigation.

## Considerations and limitations to implementing isotope labelling in AM fungal research

This section includes several discussion points regarding the use of isotopes and hyphal compartments in the lab and in the field. It is intended to serve as contextual information for the research described in this research review, as well as information for those new to the technique who are interested in using isotope labelling in their future research.

As with all botanical research, there are limitations to conducting soil-based experiments in pots or equivalent plant growth containers rather than in the field, because i) rooting volume is limiting to different degrees and possibly confounding, and ii) the results with pots are not easily extrapolated and relatable to real-world systems, both natural and agroecological. Studies with AM fungi and isotope labelling/hyphal compartments are no exception. We generally accept that there are ‘artefacts’ associated with the pot study work cited in this review and indeed broadly across plant science research. These artefacts include the presence or absence of an interacting soil microbiome—depending on whether the soil was sterilised or not, the use of small pots that restrict and modify root proliferation and thus AM fungal colonisation and function, temperature, and growth conditions in controlled environment rooms. A valuable compromise is the use of intact soil cores (e.g. Elliott et al. (2021)), which have endured relatively little manipulation, so maintain natural soil biological and physicochemical characteristics, and often involve greater soil volume than a nursery pot or PVC pipe typically used in controlled-environment studies.

Work in the field does however pose constraints on the treatments that can be implemented and controlled, especially climatic variables such as rainfall, temperature and atmospheric CO_2_, which are important for progressing research into the effects of climate change on AM associations. This issue extends to climate change research on plants and ecology broadly, of course, and researchers are continually developing ways to study plants in future climate scenarios realistically. For example, free-air carbon dioxide enrichment (FACE) facilities are semi-permanent structures that allow for atmospheric CO_2_ concentrations to be manipulated (elevated) in a defined space, either over natural or agricultural ecosystems. For example, the FACE facility in Australia (EucFACE) has been used to study the effects of elevated CO_2_ on AM and ectomycorrhizal fungi in a grassy woodland (Castañeda-Gómez et al. 2021).

There are safety and regulatory constraints on field work involving radioisotopes, due to transport of hazardous material to and from the lab in soil and plant samples, as well as the need to ensure proper containment of radioactivity in the environment (soil). Another issue that pertains to both lab and the field is the various half-lives of different radioisotopes—some allow experiments to run as long as desired, but others require a limited duration in order for the decaying radioisotope to be detected by scintillation counting in plant tissues. For example, ^33^P and ^32^P are β-emitters and have a half-life of 25.34 and 14.26 days, respectively, meaning soil needs to be dosed with substantial amounts of the isotope to allow for a typical 45–60-day experiment plus sample processing time. On the other hand, ^65^Zn is a γ-emitter and its half-life is a relatively lengthy 244 days. But γ-emitters have extra safety issues compared to β-emitters, most notably requiring lead shielding (compared to lighter and cheaper Perspex for β-emitters), reflecting the unpredictable scattering and high penetration power of γ rays. Stable isotopes, by their very nature, do not impose the same time constraints as radioisotopes because they do not decay and are relatively non-hazardous to handle. Although there are no stable isotopes of P suitable for use in plant research, there are for N, as well as a commercially available stable isotope of Zn (^67^Zn) that could be explored as safe option for future hyphal compartment research.

Another ongoing matter for discussion is how best to use hyphal compartments and isotope labelling to estimate the activity of the MUP. In the work cited in this review, almost all authors engineered the hyphal compartments differently, added isotopes differently to soil (with or without buffer soil), and perhaps most importantly reported the acquisition of resource by the MUP differently, with different calculations to reach those values. Early studies report MUP activity as Becquerels present in plant tissue, or % of isotope added that appeared in the plant material, compared between different treatments (relative quantification). Subsequently, researchers implemented equations to provide useful estimates such as % P or total amount P that arrived through the mycorrhizal versus the direct pathway of uptake (absolute quantification) (Smith et al. 2003, 2004). Of course, those are approximate estimations because the volume/mass of labelled soil accessible only to AM hyphae has been very small compared to the volume/mass of soil in the whole pot volume. To extrapolate to the whole pot, the equation takes the ratio of labelled P *vs* amount of ‘plant-available’ P in the hyphal compartment (the soil's specific activity), compared to the ratio of radioactive *vs* stable P in plant tissue (the plant's specific activity), then multiplies that value by the whole pot volume. This calculation can lead to over- or under-estimation of the actual MUP activity, because we assume that what happens in the small hyphal compartment also happens uniformly throughout the whole pot, which is highly unlikely. This is because the hyphal compartment can act as a ‘patch’ in the soil due to both its enrichment with a given nutrient through the isotope labelling process and its freedom from competition for nutrient uptake by roots. In an attempt to mitigate some of this hyphal compartment error, a ‘normalisation factor’ based on the measured hyphal length density in the hyphal compartment compared with the rest of the pot was proposed and discussed by Smith et al. (2004). However, the necessity of such a normalisation factor appears to be dependent on the AM fungal species in question, as some species develop more poorly or more extensively in the hyphal compartment while others develop uniformly in the hyphal compartment and root compartment (Drew et al. 2003; Cavagnaro et al. 2005). For example, Nagy et al. (2009) assumed that in their study, proliferation of *R. irregularis* hyphae were uniform in the hyphal compartment and root compartment (based on previous work), and thus did not include hyphal length density values in their equations.

The specific activity equations commonly used also create issues when the method used to determine ‘plant-available’ nutrient concentration in soil is not robust, for example for heavy metals such as Zn, Fe and Mn, or when different methods are used by different labs, e.g. Olsen, Colwell or resin-extractable P. ‘Plant-available’ nutrients in soils are believed to be in a form that can be transported into the plant without transformation to a different chemical form. For example, P fertiliser quickly becomes ‘fixed’ in soils by conversion to forms that are not readily taken up by root inorganic phosphate transporters, leaving a small proportion that remains ‘plant-available’. The ‘plant-available’ nutrients in a specific soil can be quantified approximately after extraction using a chemical reagent such as those mentioned above, in the case of P. There is no precise measure of plant-available Zn, and the best estimate is made using a DTPA-extract, which is known to over-estimate plant-availability. This issue was discussed by Watts-Williams et al. (2015a, b), who presented a range of Zn potentially contributed by the MUP to tomato shoots, between the value calculated using DTPA-extractable Zn, and the value calculated using a correction factor for DTPA-extractable Zn.

## Future perspectives

Some of the most pressing challenges that society faces in the next decades relate to the changing climate, which will include hotter and drier conditions in some agriculture-intensive areas. Thus, the research focus should be increasingly concentrated on how we can leverage the AM fungal association to meet sustainability goals such as food security, drought, temperature and salinity resilience, and engineering nutritious foods (Thirkell et al. 2017, 2022; Pickles et al. 2020). Timely research questions regarding the function of AM fungal resource transport could focus on:

i) Working towards accountable commercial AM fungal inoculum products that achieve reproducible and reliable results (within reason, e.g. with a certain crop) to improve P uptake efficiency and agricultural yields with reduced fertiliser—Many commercial inoculum products may not contain viable propagules (Salomon et al. 2022), so this would involve developing a quality control framework to which companies must be accountable (Pickles et al. 2020). Reporting the activity of the MUP is one way to demonstrate that an inoculum product is functioning (see Elliott et al. 2021).
ii) Finding solutions for the global challenges we face in consequence of the changing climate—There is a need for additional studies, including field-based experiments, that comprise multiple interactive variables relevant to climate change (temperature, CO_2_, water) and isotope labelling to understand how effective AM fungi will be at delivering water, P and N to plants and across mycelial networks in an increasingly hostile climate.
